# Prognostic role of the updated Kiel classification in canine high‐grade T‐cell lymphomas

**DOI:** 10.1002/vms3.1398

**Published:** 2024-05-20

**Authors:** Urszula Jankowska, Dariusz Jagielski, Michał Czopowicz, Rafał Sapierzyński

**Affiliations:** ^1^ Białobrzeska Veterinary Clinic Warsaw Poland; ^2^ Department of Diagnostics and Clinical Sciences Institute of Veterinary Medicine, Faculty of Biological and Veterinary Sciences Nicolaus Copernicus University Torun Poland; ^3^ Division of Veterinary Epidemiology and Economics Institute of Veterinary Medicine Warsaw University of Life Sciences (SGGW) Warsaw Poland; ^4^ Division of Animal Pathology Department of Pathology and Veterinary Diagnostics Institute of Veterinary Medicine Warsaw University of Life Sciences (SGGW) Warsaw Poland

**Keywords:** cytopathology, dogs, fine‐needle biopsy, high‐grade lymphoma, Kiel classification, lymphoma

## Abstract

**Background:**

The aim of this study was to determine and describe the prognostic role of the morphological subtype determined according to the updated Kiel classification in dogs with high‐grade T‐cell lymphomas (HGTCLs) depending on the treatment applied.

**Objectives:**

The HGTCLs were classified into three subtypes according to the updated Kiel classification: pleomorphic mixed (PM), lymphoblastic lymphoma/acute lymphoblastic leukaemia and plasmacytoid (P). The treatment was divided into a palliative therapy (PlT) group and a chemotherapy (ChT) group.

**Methods:**

The study was conducted between 2009 and 2017, and it enrolled 58 dogs in which cytomorphological and immunocytochemistry diagnoses were HGTCL.

**Results:**

Overall survival (OS) was significantly longer in the ChT group (median OS—4 months, interquartile range [IQR] from 2 to 8 months) than in the PlT group (median OS—6 weeks, IQR from 1 week to 3 months).

In the PlT group, PM subtype and glucocorticosteroids (GCSs) treatment proved significantly and independently linked to longer OS and approximately three‐fold lower risk of death during the study period (adjusted hazard ratio [HR_adj_] = 0.26, confidence interval [CI] 95%: 0.08–0.81; *p* = 0.020 and HR_adj_ = 0.30, CI 95%: 0.11–0.77; *p* = 0.013, respectively), although due to small group size, precision of estimations was poor (wide CI 95%).

In the ChT group, >7 days elapsing between diagnosis and the beginning of chemotherapy and GCS treatment prior to chemotherapy were significantly associated with lower chance of complete remission (CR; *p* = 0.034 for both); GCS treatment prior to chemotherapy was significantly associated with shorter OS (*p* = 0.016); chemotherapy based on the modified CHOP protocol was significantly associated with higher chance of CR (*p* = 0.034) and longer OS (*p* = 0.039); and CR was significantly linked to longer OS (*p* = 0.001).

**Clinical significance:**

The morphological subtype of HGTCL has some prognostic value in dogs treated palliatively (with PM subtype associated with longer OS than P subtype); however, this effect is no longer visible when a dog is treated with chemotherapy.

## Graphical abstract

The morphological subtype of high‐grade T‐cell lymphoma has some prognostic value in dogs treated palliatively (with pleomorphic mixed subtype associated with longer overall survival than plasmacytoid subtype); however, this effect is no longer visible when a dog is treated with chemotherapy. This observation may have some practical value. Certain factors may influence dog owners to opt out of chemotherapy; therefore, the knowledge of factors that positively affect the outcome in palliatively treated patients may turn out to be valuable.

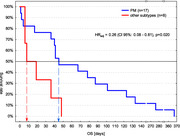

## 1 INTRODUCTION

Canine T‐cell lymphomas (TCLs) are a heterogeneous group of neoplasms, with different biologic behaviour and response to treatment. For a long time, dogs with TCL have been thought to be at significantly higher risk of worse therapeutic response, relapse and early death following therapy, compared to those with B‐cell lymphoma (Brodsky et al., 2009; Fournel‐Fleury et al., 1997; Fournel‐Fleury et al., 2002; Modiano et al., 2005; Ponce et al., 2004; Ruslander et al., 1997; Sueiro et al., 2004; Teske et al., 1994; Valli et al., 2013; Wilkerson et al., 2005). However, TCL may be classified into high‐grade TCL (HGTCL) and low‐grade TCL (e.g. T‐zone lymphoma), with the latter having an indolent course (Seelig et al., 2014). Therefore, using the immunophenotype as the only prognostic factor does not seem to be sufficient (Ponce et al., 2004; Valli et al., 2006). According to the Revised European‐American Classification of Lymphoid Neoplasms and World Health Organisation (WHO), the current classification of canine lymphomas is based on cytologic/histologic parameters (morphotype, i.e., cell morphology and lymph node tumour architecture), immunophenotype (B or T), cytogenetic profile, clinical and epidemiological data (Campo et al., 1998; Harris et al., 1994). The morphologic classification based on the updated Kiel classification adapted to the canine species has generally proven to have some prognostic significance in canine lymphomas (Ponce et al., 2004; Sayag et al., 2018). In this study, we decided to focus on the prognostic role of the morphological subtype determined according to the updated Kiel classification in dogs with HGTCL depending on the treatment applied.

## 2 MATERIALS AND METHODS

### 2.1 Study population

The study was conducted between 2009 and 2017, and it enrolled 58 dogs in which cytomorphological diagnosis of the high‐grade lymphoma was made and identified using the immunocytochemistry as HGTCL twice by the same clinical cytologist (the period between first examination and re‐examination of slides was 1–2 months). Demographic and clinical data for these dogs were collected. Demographic data included age, breed, sex and neuter status. Clinical data included the results of: (i) clinical examination; (ii) abdominal ultrasonography; (iii) thoracic radiography in two projections (lateral and dorso‐ventral); (iv) complete blood count including total and differential white blood cell count, red blood cell count, haemoglobin concentration, haematocrit and platelet count; (v) blood biochemistry including the concentration of total protein, albumin, total bilirubin, creatinine, urea and total calcium (tCa) corrected for albumin concentration (Ca_corr_) according to the following formula: Ca_corr_ [mmol/L] = tCa [mmol/L] + 0.02 × (40 – albumin [g/L] (Payne et al., 1973), as well as the activity of alanine aminotransferase, aspartate aminotransferase and alkaline phosphatase; (vi) the information about the treatment protocol applied; and (vii) the information about the outcome.

### 2.2 Lymphoma diagnosis

Definitive diagnosis of HGTCL was based on cytology followed by immunocytochemistry. Smears for cytological examination were obtained by a fine‐needle aspiration or non‐aspiration biopsy (FNB) from enlarged lymph nodes (in all dogs; in cases of systemic lymphadenomegaly, at least three samples were collected from at least two enlarged lymph nodes) and from additional sites, including enlarged internal organs (splenomegaly in 40 dogs), abnormal masses located on the skin (in one dog) and in the body cavities (mediastinal tumours in five dogs), bone marrow with atypical cells in peripheral blood in five dogs, depending on the disease presentation. For routine examination, at least three smears of aspirates were used. The smears were dried, fixed in 70% methanol, stained with Giemsa solution, and examined by light microscopy (Olympus CX21).

The immunophenotype of lymphoma (B cell or T cell) was determined using immunocytochemistry. Briefly, smears from each dog were dried, fixed in acetone at 4°C for 5–10 min and stained immediately or stored at −20°C. Immunocytochemical staining was performed according to Caniatti et al. (1996) and Sapierzyński (2010) using commercially available antibodies (Dako) for the pan‐T‐lymphocyte marker CD3 (polyclonal rabbit anti‐human) and B‐cell antigen receptor complex CD79α (monoclonal mouse anti‐human). Two smears from the same case were stained using both antibodies. The intensity of expression of examined CD antigens in cytological preparations was determined in the light microscope, and the result was considered positive if at least 80% of large or medium‐sized lymphocytes showed a strong cytoplasmic reaction. CD3 and CD79α immunopositive cells were counted from 1000 cells using an Olympus BX41 microscope coupled to a computer equipped with a CellA analysis system (images of microscopic fields abundant in well‐preserved cells were captured and formatted as TIFF files, and immune expression of CD3 and CD79α was calculated). A TCL immunophenotype was identified if at least 80% of lymphomatous cells were CD3‐immunopositive. Negative controls were processed in the same manner, using a buffer solution instead of primary antibodies. The positive controls for CD3 and CD79α were cellular samples collected from impression smears of canine hyperplastic lymph node. Immunocytochemistry results were interpreted without information from routine cytology.

The morphotype (high‐ and low‐grade) and subtype of TCL were determined based on the updated Kiel classification adapted for dogs as previously described (Fournel‐Fleury et al., 1997; Fournel‐Fleury et al., 2002; Ponce et al., 2010). The following morphological features of cells were evaluated: size and shape of cells, volume and intensity of cytoplasm staining, size and shape of nuclei, the position of nucleus in a cell; size, distinctness, number and positioning of nucleoli and appearance of nuclear chromatin. Additionally, the mitotic rate in cytological specimens was established according to Meuten et al. (2015). HGTCL was recognised if at least 80% of medium and/or large cells and moderate or high mitotic count (MC) were observed. MC was estimated in cytological smears by using an ocular FN 22 mm (most common) the area of a ‘high power (40×) field’ = 0.237 mm^2^. A low MC was defined as 0–2 mitosis/ fields, medium as 3 to 5/fields and high as 6 or more mitoses/fields (Ponce et al., 2003). The HGTCLs were further classified into three morphological subtypes according to the updated Kiel classification: pleomorphic mixed (PM), lymphoblastic lymphoma/acute lymphoblastic leukaemia (LB), and plasmacytoid (P).

### 2.3 Clinical staging

Dogs were staged according to the WHO V‐stage criteria for canine lymphomas (Owen, 1980). Dogs were classified as a Clinical Stage I if a single lymph node was enlarged, Stage II—if multiple lymph nodes on one side of the diaphragm were enlarged, Stage III—if multiple lymph nodes on both sides of the diaphragm were enlarged but without hepato‐ or splenomegaly on abdominal ultrasonography, Stage IV—if hepato‐ or splenomegaly was found on abdominal ultrasonography, and Stage V—if lymphoma cells were found in bone marrow aspirates. Furthermore, dogs were assigned to substage categories of ‘a’ (without apparent systemic clinical signs) or ‘b’ (with apparent systemic clinical signs). Given that most dogs did not have bone marrow analysis, for the needs of statistical analysis, a more general dichotomous classification into Stages I–III and Stages IV and V was applied. The mediastinal form was classified as Stage II unless bone marrow involvement was detected, and then the dog was assigned to Stage V.

### 2.4 Treatment

The dogs were categorised based on the treatment applied into two groups: the palliative therapy (PlT) group in which only supportive or glucocorticosteroid (GCS) therapy was used and the chemotherapy (ChT) group in which chemotherapeutic agents were used. Supportive therapy included non‐steroid anti‐inflammatory agents and antibiotics if necessary, as well as a special diet. GCS treatment in the PlT group was based on oral prednisolone 1 mg/kg BID. In the ChT group, chemotherapeutic protocols were divided into basic (COP—cyclophosphamide, vincristine, prednisolone; or CHOP—cyclophosphamide, doxorubicin, vincristine, prednisolone; or single chemotherapeutic agents) and modified (CHOP with L‐asparaginase and/or lomustine; custom treatment schemes). GCS pre‐treatment was defined as any use of GCS (oral or injectable) between FNB and the onset of chemotherapy but exceeding a week's treatment.

### 2.5 Outcome

The outcome was defined as the overall survival time (OS) in the PlT group and as the achievement of complete remission (CR) and OS in the ChT group. OS was the time from the day of FNB based on which the final diagnosis of HGTCL was made through the day of death. No observations were censored since all dogs were followed until death, and all deaths were considered as related to HGTCL. CR was defined as complete resolution of all signs of disease, including normal‐size lymph nodes. Partial remission was defined as >30% reduction in the mean longest dimension of lesions (Vail et al., 2010), based on the assessment of the size of lymph nodes registered in the medical records.

### 2.6 Statistical analysis

Numerical variables were presented as the median, interquartile range (IQR) and range and compared between groups using the Mann–Whitney U test (two groups) or Kruskal–Wallis H test (three groups). Categorical variables were expressed as the count and percentage of cases in a group and compared between groups using the maximum likelihood G test or Fisher exact test, if the expected count in any cell of the contingency table was below 5. The Wilson score method was used to work out 95% confidence interval (CI 95%) for proportions (Altman et al., 2000). The link between the HGTCL subtype and the outcome was analysed according to a two‐level procedure separately in the PlT and ChT groups. First, the univariable analysis was carried out using the univariable Cox proportional‐hazard model (OS analysis) or simple logistic regression (CR analysis); the magnitude of the effect was reported as the crude hazard ratio (HR_crude_) or the crude odds ratio (OR_crude_), respectively. As OS was exactly known for all dogs and all deaths were considered as related to HGTCL, there were no censored observations in the survival analysis. If the HGTCL subtype proved significantly linked with the outcome, the multivariable analysis was run including the morphological subtype of HGTCL, and these demographic and clinical variables had *p* < 0.1 in the univariable analysis. The multivariable analysis was performed using the multivariable Cox proportional‐hazard model with a backwards stepwise elimination procedure, and adjusted hazard ratios (HR_adj_) were reported. OS was graphically presented using the Kaplan–Meier plots. All statistical tests were two‐sided. A significance level (*α*) was set at 0.05 except for the univariable analysis in which *α* = 0.1. The statistical analysis was performed in TIBCO Statistica 13.3 (TIBCO Software Inc.).

## 3 RESULTS

### 3.1 Study population

The study population consisted of 58 dogs with HGTCL—30 males (nine neutered) and 28 females (15 spayed), aged 1 to 14 years with a median (IQR) of 7 (6–8) years. Forty‐seven dogs (81%) were pedigreed with 18 boxers, followed by Dogue de Bordeaux (six dogs), American Staffordshire terrier (three dogs), French bulldog, dachshund, beagle and golden retriever (two dogs each), and German shepherd, Irish setter, Caucasian sheepdog, west highland white terrier, giant schnauzer, Saint Bernard, Bernese mountain dog, Central Asian shepherd, English bulldog, Irish wolfhound, Newfoundland and standard schnauzer (one dog each). Based on the updated Kiel classification 44/58 dogs (76%, CI 95%: 64%–85%) had PM, 8/58 had P (14%, CI 95%: 7%–25%) and 6/58 (10%, CI 95%: 5%–21%) had LB (Table 1). According to the WHO classification, the first two subtypes (PM and P) corresponded to the peripheral TCL not otherwise specified, and the latter subtype (LB) corresponded to the precursor TCL/leukaemia. There were no discrepancies in the lymphoma classification between the first and the second examination of slides nor between plain microscopic examination and immunophenotyping. Multicentric lymphoma was present in 52/58 dogs (90%, CI 95%: 79%–95%) and 5/58 dogs (9%, CI 95%: 4%–19%) had the mediastinal form (three PM and two P types; Table 1). One dog (an 8‐year‐old spayed female boxer) had also signs of cutaneous involvement (PM subtype) and it was also the dog that survived the longest time (367 days) without chemotherapy, only on GCS. On presentation, clinical signs were observed in 50/58 dogs (86%, CI 95%: 75%–93%), including all dogs with the mediastinal form and one dog with the cutaneous form of HGTCL (Table 1). The most common signs were apathy (35/50, 70%), lack of appetite (29/50, 58%), weight loss (18/50, 36%), polyuria/polydipsia (15/50, 30%), exercise intolerance (13/50, 26%) and vomiting (12/50, 24%). Hypercalcemia (Ca_corr_ >3.0 mmol/L) was present in 10/47 dogs for which Ca measurement was available (21%, CI 95%: 12%–35%), including 3/5 dogs with the mediastinal form. According to what the owners declared, the time that elapsed between the onset of the first symptoms and the diagnosis ranged from 1 to 194 days with a median (IQR) of 21 (14–37) days (Table 1). Demographic and clinical characteristics of dogs with different HGTCL subtypes did not differ significantly. Due to the small number of dogs with the P and LB subtypes, they were merged into one group and the further analyses involved comparisons between PM and non‐PM dogs.

**TABLE 1:** Demographic and clinical characteristics of dogs with high‐grade T‐cell lymphoma (HGTCL) enrolled in the study with division into two treatment groups.

		Therapy group		
Variable	Overall (*n* = 58)	PlT (*n* = 23)	ChT (*n* = 35)	*p*‐value
**Demographic characteristics**				
Age [years] ^a^	7, 6–8 (1–14)	8, 6–10 (5–14)	7, 5–8 (1–12)	0.057
Sex and neuter status				0.804
ME	21 (36%)	9 (39%)	12 (34%)	
MN	9 (16%)	3 (13%)	6 (17%)	
FE	13 (22%)	4 (17%)	9 (26%)	
FN	15 (26%)	7 (31%)	8 (23%)	
Pedigree dogs	47 (81%)	20 (87%)	27 (77%)	0.499
Boxer	18	10	8	
Dog de Bordeaux	6	3	3	
American Staffordshire terrier	3	1	2	
French bulldog	2	0	2	
Dachshund	2	1	1	
Beagle	2	0	2	
Golden retriever	2	1	1	
German Shepherd	1	1	0	
Irish setter	1	1	0	
Caucasian sheepdog	1	0	1	
West Highland White terrier	1	0	1	
Giant schnauzer	1	0	1	
Standard schnauzer	1	1	0	
Saint Bernard dog	1	0	1	
Bernese mountain dog	1	0	1	
Central Asian shepherd dog	1	0	1	
English bulldog	1	0	1	
Irish wolfhound	1	0	1	
Newfoundland	1	1	0	
**Clinical characteristics**				
Time between first symptoms observed by the owned and HGTCL diagnosis [days] ^a^	21, 14–37 (1–194)	21, 13–48 (1–194)	21, 14–35 (1–120)	0.594
Clinical form				0.390
Multicentric	52 (90%)	20 (87%)	32 (91%)	
Mediastinal	5 (8%)	2 (9%)	3 (9%)	
Cutaneous	1 (2%)	1 (4%)	0	
Clinical stage [b substage]				0.610
II	9 (15%) [7]	3 (13%) [3]	6 (17%) [4]	
III	10 (17%) [7]	7 (30%) [5]	3 (9%) [2]	
IV	34 (59%) [31]	10 (44%) [10]	24 (68%) [21]	
V	5 (9%) [5]	3 (13%) [3]	2 (6%) [2]	
**Cytological characteristics**				
WHO classification				0.386
Unspecified peripheral TCL	52 (90%)	22 (96%)	30 (86%)	
Precursor TCL/leukaemia	6 (10%)	1 (4%)	5 (14%)	
Kiel classification				0.201
PM	44 (76%)	17 (74%)	27 (77%)	
LB	6 (10%)	1 (4%)	5 (14%)	
P	8 (13%)	5 (22%)	3 (9%)	
OS [days]^a^	94, 37–211 (1–371)	41, 8–87 (1–367)	123, 65–237 (7–371)	0.001

Abbreviations: ChT, chemotherapy; FE, female entire; FN, female neutered; LB, lymphoblastic lymphoma/acute lymphoblastic leukaemia; ME, male entire; MN, male neutered; OS, overall survival; P, plasmacytoid; PlT, palliative therapy; PM, pleomorphic mixed; WHO, World Health Organisation.

^a^ Presented as the median, interquartile range (IQR) and range.

**FIGURE 1:**
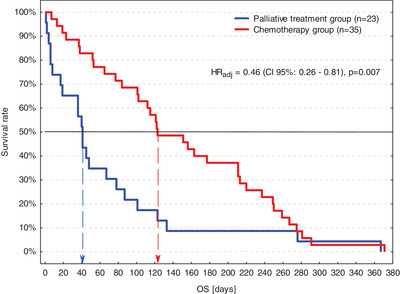
Kaplan–Meier survival curves of dogs with high‐grade T‐cell lymphoma (HGTCL) treated or not with chemotherapy. CI 95%, 95% confidence interval; HR_adj_, adjusted hazard ratio; OS, overall survival time.

Of 58 dogs with HGTCL, 23 (40%) underwent PlT (PlT group) and 35 (60%) underwent chemotherapy (ChT group). Neither the distribution of HGTCL subtypes (*p* = 0.201) nor the demographic and clinical characteristics of dogs differed significantly between the PlT and ChT groups (Table 1). Controlled for the clinical stage on presentation and HGTCL subtype, OS was significantly longer in the ChT group (median OS–4 months, IQR–2 to 8 months) than in the PlT group (median OS–6 weeks, IQR–1 week to 3 months; *p* = 0.007; Table 2, Figure 1). Therefore, the prognostic role of the HGTCL subtype was further analysed separately in the PlT and ChT groups (Table 3). Demographic and clinical characteristics of dogs did not differ significantly between PM and non‐PM dogs in either group (Table 3); however, due to small group sizes, the power of these comparisons was poor, and therefore all variables were included in the univariable OS and CR analyses to avoid missing potential confounding factors.

**TABLE 2:** The multivariable Cox proportional hazard model showing the effect of chemotherapy on the risk of death of high‐grade T‐cell lymphoma (HGTCL) controlled for clinical stage and subtype according to updated Kiel classification.

Variable	Regression coefficient SE	χ^2^ statistic	*p*‐value	HR_adj_ (CI 95%)
Chemotherapy	−0.79 (0.29)	7.21	0.007	0.46 (0.26–0.81)
Clinical stage				
II/III	Reference category			
IV/V	0.20 (0.31)	0.43	0.510	1.23 (0.67–2.24)
Kiel classification				
PM	Reference category			
LB	−0.17 (0.46)	0.14	0.705	0.84 (0.34–2.06)
P	0.78 (0.41)	3.68	0.055	2.18 (0.98–4.82)

Abbreviations: CI 95%, 95% confidence interval; HR_adj_, adjusted hazard ratio; LB, lymphoblastic lymphoma/acute lymphoblastic leukaemia; OS, overall survival; P, plasmacytoid; PM, pleomorphic mixed; SE, standard error.

**TABLE 3:** Demographic and clinical characteristics of dogs in PlT and ChT groups depending on the morphologic subtype of HGTCL according to the updated Kiel classification.

	PlT group (*n* = 23)			ChT group (*n* = 35)		
	Subtype according to Kiel classification					
Variable	PM (*n* = 17)	Non‐PM (*n* = 6)	*p*‐value	PM (*n* = 27)	Non‐PM (*n* = 8)	*p*‐value
Age [years]^a^	8, 7–9 (5–13)	6 (6–14)	0.749	7, 4–8 (1–14)	8, 6–10 (5–12)	0.081
Sex and neuter status			0.829			0.169
ME	6 (35%)	3 (50%)		9 (33%)	3 (38%)	
MN	2 (12%)	1 (17%)		4 (15%)	2 (25%)	
FE	3 (18%)	1 (17%)		6 (22%)	3 (38%)	
FN	6 (35%)	1 (17%)		8 (30%)	0	
Clinical stage IV/V	9 (53%)	5 (83%)	0.340	20 (74%)	6 (75%)	0.999
GCS treatment^b^	11 (65%)	4 (67%)	0.999	10 (37%)	3 (38%)	0.999
Chemotherapy protocol	–	–	–			0.332
Basic				11 (41%)	2 (25%)	
Modified				16 (59%)	6 (75%)	

Abbreviations: ChT, chemotherapy; FE, female entire; FN, female neutered; GCS, glucocorticosteroid; ME, male entire; MN, male neutered; PlT, palliative therapy; PM, Pleomorphic mixed.

^a^ Presented as the median, interquartile range (IQR) and range.

^b^ Prior to chemotherapy in the ChT group.

### 3.2 PlT group

**FIGURE 2:**
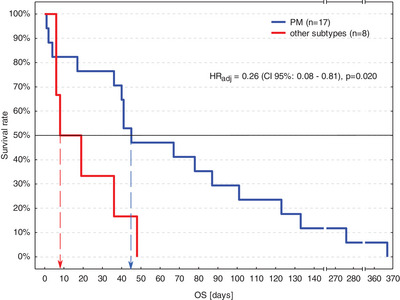
The Kaplan–Meier survival curves of dogs with HGTCL treated palliatively with pleomorphic mixed (PM) and other subtypes according to Kiel classification. Arrows indicate median overall survival time (OS) for each group. CI 95%, 95% confidence interval; HR_adj_, adjusted hazard ratio.

Seventeen out of 23 dogs (74%) had the PM subtype and 6/23 dogs (26%) had the non‐PM subtype of HGTCL (five P and one LB); 8/23 dogs (35%) received only supportive therapy, while 15/23 dogs (65%) were treated with GCS. PM subtype and two other variables (GCS treatment and being a neutered female) had *p* < 0.1 in the univariable survival analysis (Table 4) and were entered into the multivariable survival analysis. Of them PM subtype and GCS treatment proved significantly and independently linked to longer OS (Figure 2) and approximately three‐fold lower HR_adj_, although due to small group size precision of estimations was poor (wide CI 95%; Table 5).

**TABLE 4:** The relationship between demographic and medical characteristics of dogs in the PlT and ChT group and their OS times.

Variable	Category	OS [days]^a^	HR_crude_ (CI 95%)	*p*‐value
**Palliative group**				
Age [years]	–	–	0.98 (0.80–1.18)	0.772
Sex and neuter status	ME (*n* = 9)	17, 6–41 (2–133)	Reference category	
	MN (*n* = 3)	4 (8–78)^b^	0.91 (0.24–3.51)	0.901
	FE (*n* = 4)	29 (1–87)^b^	1.15 (0.34–3.85)	0.820
	FN (*n* = 7)	67, 45–276 (36–367)	0.34 (0.11–1.01)	0.054^*^
Clinical stage	IV/V (*n* = 14)	42, 8–87 (1–276)	1.08 (0.44, 2.59)	0.869
	II/III (*n* = 9)	41, 36–45 (4–367)		
GCS treatment	Yes (*n* = 15)	48, 36–123 (6–367)	0.34 (0.13–0.85)	0.021^*^
	No (*n* = 8)	6, 4–40 (1–101)		
Morphological subtype	PM (*n* = 17)	45, 36–101 (1–367)	0.31 (0.11–0.91)	0.033^*^
	Non‐PM (*n* = 6)	8, 6–36 (6–48)		
**Chemotherapy group**				
Age [years]	–	–	1.08 (0.93–1.25)	0.324
Sex and neuter status	ME (*n* = 12)	136, 89–170 (37–259)	Reference category	
	MN (*n* = 6)	249, 220–281 (13–371)	0.30 (0.10–0.91)	0.033
	FE (*n* = 9)	123, 84–211 (7–291)	0.63 (0.25–1.56)	0.319
	FN (*n* = 8)	88, 30–179 (19–275)	0.99 (0.40–2.48)	0.985
Clinical stage	IV/V (*n* = 26)	136, 65–220 (7–291)	1.65 (0.73–3.72)	0.225
	II/III (*n* = 9)	123, 112–267 (37–371)		
Time between diagnosis and the beginning of chemotherapy	>7 days (*n* = 13)	123, 101–249 (37–281)	0.96 (0.48–1.94)	0.920
	≤7 days (*n* = 22)	136, 52–220 (7–371)		
GCS treatment prior to chemotherapy	Yes (*n* = 15)	112, 53–151 (13–213)	2.68 (1.20–5.95)	0.016
	No (*n* = 8)	170, 84–259 (7–371)		
Chemotherapeutic protocol	Modified (*n* = 22)	177, 112–250 (53–371)	0.48 (0.23–0.96)	0.039
	Basic (*n* = 13)	52, 23–123 (7–291)		
CR	Yes (*n* = 13)	237, 177–267 (122–371)	0.28 (0.13–0.60)	0.001
	No (*n* = 22)	92, 38–123 (7–281)		
Morphological subtype	PM (*n* = 27)	123, 52–237 (7–371)	1.40 (0.62–3.13)	0.419
	Non‐PM (*n* = 8)	167, 108–250 (53–291)		

Abbreviations: ChT, chemotherapy; CI 95%, 95% confidence interval; CR, complete remission; FE, female entire; FN, female neutered; GCS, glucocorticosteroid; HR_crude_, crude hazard ratio; ME, male entire; MN, male neutered; OS, overall survival time; PlT, palliative therapy, PM, Pleomorphic mixed.

^a^ Presented as the median, interquartile range (IQR) and range in parentheses.

^b^ Only median and range presented due to small number of dogs.

* Included in the multivariable analysis.

**TABLE 5:** The multivariable Cox proportional hazard model showing the effect of variables on the risk of death of HGTCL in the PlT group.

Variable	Regression coefficient SE	χ^2^ statistic	*p*‐value	HR_adj_ (CI 95%)
PM subtype	−1.34 (0.58)	5.44	0.020	0.26 (0.08–0.81)
GCS treatment	−1.21 (0.48)	6.18	0.013	0.30 (0.11–0.77)
Variables dropped from the final model:				
Neutered females	−0.85 (0.59)	2.12	0.145	0.43 (0.14–1.34)

Abbreviations: CI 95%, 95% confidence interval; HR_adj_, adjusted hazard ratio; SE, standard error.

### 3.3 ChT group

Twenty‐seven out of 35 dogs (77%) had the PM subtype and 8/35 (23%) had the non‐PM subtype of HGTCL (3 P and 5 LB). The time that elapsed between FNB and the beginning of chemotherapy ranged from 1 to 98 days with a median (IQR) of 5 (3−15) days (in 13 dogs, this time exceeded 1 week and in five dogs—1 month). Within this time, 13/35 dogs (37%) received GCS; 13/35 dogs (37%) were treated according to the basic chemotherapeutic protocol and 22/35 (63%) according to the modified CHOP protocol. CR was achieved in 13/35 dogs (37%, CI 95%: 23%–54%) and lasted from 41 to 341 days with the median (IQR) of 103 (77–142) days. Partial remission was achieved in 10 dogs (29%, CI 95%: 16%–45%). Median OS (range) was 123 (7–371) days for PM, 213 (53–291) days for LB, and 121 (115–220) days for P. PM subtype did not prove to be significantly linked to CR (OR_crude_ = 2.1, CI 95%: 0.4–3.8; *p* = 0.680; Table 6) or OS (HR_crude_ = 1.4, CI 95%: 0.6–3.1; *p* = 0.419; Table 4), so multivariable analysis was not performed. However, at such a small sample size, the probability of a type II (β) error (i.e., falsely insignificant result) was very high (85%−90%), so these negative results should be treated with high caution. The univariable analysis also showed that >7 days elapsing between diagnosis and the beginning of chemotherapy and GCS treatment prior to chemotherapy were associated with a significantly lower chance of CR (*p* = 0.034 for both). Additionally, at least 7 days of GCS treatment prior to chemotherapy was associated with shorter OS (*p* = 0.016); also chemotherapy based on the modified CHOP protocol was associated with a significantly higher chance of CR (*p* = 0.034) and longer OS (*p* = 0.039), and CR was linked to significantly longer OS (*p* = 0.001) (Tables 4 and 6).

**TABLE 6:** The relationship between demographic and medical categorical characteristics of dogs in chemotherapy group and the occurrence of CR.

Variable	Category	CR/all dogs in the category (%)	OR_crude_ (CI 95%)	*p*‐value
Age	–	–	1.09 (0.83–1.44)	0.530
Sex and neuter status	ME (*n* = 12)	4 (33.3)	Reference category	0.497
	MN (*n* = 6)	3 (50.0)	2.00 (0.27–14.8)	
	FE (*n* = 9)	3 (33.3)	1.00 (0.16–6.26)	0.999
	FN (*n* = 8)	3 (37.5)	1.20 (0.19–7.77)	0.848
Clinical stage IV/V	Yes (*n* = 26)	10 (38.5)	1.25 (0.25–6.16)	0.999
	No (*n* = 9)	3 (33.3)		
Time between diagnosis and the beginning of chemotherapy	>7 days (*n* = 13)	2 (15.4)	0.18 (0.04–0.93)	0.034
	≤7 days (*n* = 22)	11 (50.0)		
GCS treatment prior to chemotherapy	Yes (*n* = 13)	2 (15.4)	0.18 (0.04–0.93)	0.034
	No (*n* = 22)	11 (50.0)		
Chemotherapeutic protocol	Modified (*n* = 22)	11 (50.0)	5.50 (1.08–28.1)	0.034
	Basic (*n* = 13)	2 (15.4)		
Morphologic subtype	PM (*n* = 27)	11 (40.7)	2.06 (0.35–12.2)	0.680
	Non‐PM (*n* = 8)	2 (25.0)		

Abbreviations: CI 95%, 95% confidence interval; CR, complete remission; FE, female entire; FN, female neutered; GCS, glucocorticosteroid; ME, male entire; MN, male neutered; ORcrude, crude odds ratio; PM, pleomorphic mixed.

## 4 DISCUSSION

Our study provided one obvious and one more sophisticated conclusion. The former is that any chemotherapy significantly extends the OS of dogs with HGTCL, compared to no or sole GCS therapy. This has been clearly evidenced in previous studies (Brown et al., 2018; Brodsky et al., 2009; Morgan et al., 2018; Rebhun et al., 2011). The difference in median OS in our study was three‐fold (6 vs. 18 weeks). OS of non‐treated dogs was only slightly shorter from commonly accepted figures (Bell et al., 1984; Squire et al., 1973), probably due to some reluctance of Polish dog owners to pursue more sophisticated diagnostic procedures like FNB (if the time that elapsed from the first symptoms through FNB to death is added to OS, it corresponds to figures reported in the literature). What is surprising is a very short OS of dogs treated with chemotherapy in our study and a low proportion of CR achieved (roughly more than one‐third of dogs). This applies both to standard COP/CHOP protocol as well as more advanced protocols with L‐asparaginase and lomustine, even though the latter dogs survived significantly longer. The poor prognosis of dogs on chemotherapy may be to some extent explained by a considerably delayed beginning of chemotherapy in a high number of dogs and frequent use of GCS before entering chemotherapy. These factors are well known to negatively affect response to chemotherapy (Gavazza et al., 2009; Price et al., 1991), and this negative relationship was also apparent in our study. Nevertheless, it would be naïve not to look for the culprit in the chemotherapy or previous steroid therapy because difficulties must also be taken into account, such as irregular medication administration, or the need to modify drug doses due to increased side effects and poor general condition of dogs.

More important appears to be the second conclusion that the morphologic subtype of HGTCL has prognostic value in dogs, which are not treated with chemotherapy. Independent of the use of GCS, dogs with the PM subtype are expected to survive longer than dogs with the non‐PM subtype. This observation applies in fact to the comparison between PM and P subtype as only one non‐treated dog had the LB subtype in our study, and its OS was 48 days, which is similar to the median OS of PM dogs. Therefore, our study in fact shows that the P subtype is an unfavourable prognostic in dogs whose owners do not opt for chemotherapy. This result is not surprising as the P subtype appears to be the most aggressive of all HGTCL (Ponce et al., 2003; Sayag et al., 2018). It is important to stress that we would not be able to detect any differences between subtypes if we used the WHO classification as PM and P are grouped there together as peripheral TCL are not otherwise specified (Valli et al., 2010). This observation may have applications in clinical practice. Most of the information available concerns treated dogs, and there are not many references to untreated dogs. More data concern steroid therapy, which is an obvious truth that it improves the general condition of the animal. Many owners, for various reasons, opt out of chemotherapy. However, they want to obtain information about the course of the disease. Therefore, knowledge about factors that positively influence the prognosis in patients undergoing palliative treatment may prove valuable.

The role of morphological subtype turned out to be insignificant in dogs treated with chemotherapy. Obviously, it can be a false negative result due to the very low power of this study as a single previous study reported significant differences in OS between dogs with three subtypes of HGTCL with P associated with the shortest and PM with the longest OS (Ponce et al., 2004). This study included a similarly low number of dogs to ours (10 PM, 10 LB and eight P), so its results should also be treated with caution. Generally, no strong evidence indicating a prognostic role of HGTCL subtype in dogs undergoing chemotherapy has so far been published (Sayag et al., 2018; Zandvliet, 2016). It is possible that the influence of chemotherapy on the natural course of HGTCL is sufficiently strong to suppress any variations related to the morphologic subtype, which would be apparent if the disease was allowed to follow its natural course. This is in fact what our study implies although it is still only a hypothesis that requires further thorough prospective investigation on a markedly larger study population. At this time, rarely are non‐treated dogs the subjects of scientific studies since most current veterinary research focuses on various chemotherapeutic protocols. However, we think that our observation may have some practical value. Even though thanks to increasing owners’ awareness and the popularity of pet health insurance, currently diseased dogs have access to modern therapies (Lloyd, 2006), there will always be situations in which palliative will be chosen as a result of financial constraints or a certain system of values of owners and doctors (Gates et al., 2019). Therefore, the knowledge of factors that positively affect the outcome in palliatively treated patients may turn out to be valuable.

In our study population, we found only three morphological subtypes of HGTCL. This is fewer compared to four (pleomorphic large cell and PM cell [small and large], lymphoblastic and P) described in Fournel‐Fleury et al. (1997; nine dogs), Fournel‐Fleury et al. (2002; 33 dogs) and six (the aforementioned plus immunoblastic and aggressive large granular) in Ponce et al. (2010; 96 dogs). P subtype was found similarly often: 8/58 HGTCL (14%) in ours, 7/42 HGTCL (17%, CI 95%: 8%–31%) in Fournel‐Fleury et al. (1997) and Fournel‐Fleury et al. (2002) and 19/96 HGTCL (20%, CI 95%: 13%–29%) in Ponce et al. (2010). LB was less often found in our study (6/58, 10%) than by Fournel‐Fleury et al. (1997) and Fournel‐Fleury et al. (2002; 29%, CI 95%: 17%–44%) and in Ponce et al. (2010; 18%, CI 95%: 11%–27%). The highest discrepancy was in the PM subtype as all previous studies identified distinct pleomorphic large cell and PM cell small and large.

We did not observe any significant differences in the demographic characteristics or clinical presentation of dogs. Neither did we find any significant difference in CR or OS between dogs with I–III and IV and V stages. We decided on this division for two reasons: First, some stages were very rare in the compared groups (e.g., only one dog with PM subtype and Stage V); second and more important was that accurate differentiation between clinical stages requires a full panel of diagnostic tests including bone marrow analysis as well as more sensitive imaging diagnostic tests than radiographs and ultrasonography applied also to the central nervous system. These tests were unavailable in most of our patients, so it is quite likely that many of them would have been reclassified to a higher stage if additional diagnostics were performed (Vail et al., 2010). This especially applies to the reclassification from Stages IV to V, which is crucial as Stage V is in fact the one whose prognostic role has so far been implied (Zandvliet, 2016). Most studies to date have not found a link between clinical stages and prognosis (Bennett et al., 2023; Dobson et al., 2001; Garrett et al., 2002; Greenlee et al., 1990; Hahn et al., 1992; Keller et al., 1993; Kiupel et al., 1999; Valerius et al., 1997). Some studies found a negative prognostic role of the ‘b’ substage (Keller et al., 1993; Valerius et al., 1997), but almost all dogs in our study were ill, so we could not include this factor as a potential confounder.

Using GCS in dogs with HGTCL turned out to be significantly associated with longer OS, which agrees with current knowledge and observations in all canine lymphomas irrespective of the immune‐ or morphotype (Zandvliet, 2016). Given that GCS treatment usually improves the quality of a dog's life, its use is highly recommended in dogs whose owners do not decide on chemotherapy. It is crucial, however, to make the owners aware of the negative effect GCS treatment has on further chemotherapy if they change their mind later. Some of the dogs enrolled in our study had already been treated with GCS by a general practitioner before making a tentative diagnosis of lymphoma and referring the dog to an oncologist. Regardless of the scenario, GCS treatment prior to chemotherapy should be considered as a negative prognostic factor.

In conclusion, our study implies that the morphological subtype of HGTCL has some prognostic value in dogs treated palliatively (with the PM subtype associated with longer OS than the P subtype); however, this effect is no longer visible when a dog is treated with chemotherapy. Therefore, morphological subtyping according to the updated Kiel classification should be a routine part of the diagnostic protocol of these dogs with HGTCL whose owners do not wish to pursue chemotherapy.

## AUTHOR CONTRIBUTIONS

**Urszula Jankowska**: Conceptualisation; methodology; writing—original draft preparation; writing—review and editing. **Dariusz Jagielski**: Conceptualisation; methodology; writing—original draft preparation; writing—review and editing. **Michał Czopowicz**: Conceptualisation; methodology; data analysis; writing—review and editing. **Rafał Sapierzyński**: Conceptualisation; methodology; writing—original draft preparation; writing—review and editing.

## CONFLICT OF INTEREST STATEMENT

None of the authors of this article has a financial or personal relationship with other people or organisations that could inappropriately influence or bias the content of this paper.

## FUNDING INFORMATION

The entire amount was paid from the first author's private incomes.

### ETHICS STATEMENT

Good clinical practice guidelines for standard‐of‐care management of sick dogs were observed, and a written consent form was signed by the owners of dogs before chemotherapy.

### PEER REVIEW

The peer review history for this article is available at https://publons.com/publon/10.1002/vms3.1398

## Data Availability

The data that support the findings of this study are available on request from the corresponding author. The data are not publicly available due to privacy or ethical restrictions.
